# Lens epithelial cells senescence in cataract pathogenesis and emerging therapeutic opportunities

**DOI:** 10.3389/fcell.2026.1758898

**Published:** 2026-03-04

**Authors:** Liangning Cui, Yiyi Wang, Lijun Wang, Jianhao Bai, Wanru Zhou, Manhui Zhu, Jianjun Zhou, Haiying Jin

**Affiliations:** 1 Department of Ophthalmology, Shanghai East Hospital, School of Medicine, Tongji University, Shanghai, China; 2 Research Center for Translational Medicine, Cancer Stem Cell Institute, Shanghai East Hospital, School of Medicine, Tongji University, Shanghai, China

**Keywords:** cataract, cellular senescence, lens epithelial cells, senolytics, senomorphics

## Abstract

Lens epithelial cells (LECs) senescence is a central pathogenic mechanism in cataract formation, driven by a variety of chronic stressors such as oxidative damage, UV radiation, metabolic disturbances, and mechanical strain. When exposed to these stressors, LECs undergo a series of cellular responses, including stable cell cycle arrest, development of a senescence-associated secretory phenotype (SASP), and impaired autophagic flux. These alterations compromise the function of LECs, disrupt lens homeostasis, and promote the pro-inflammatory microenvironment that accelerates cataract progression. Recent advances in targeting senescent LECs have led to the development of promising pharmacological therapies, including senolytics and senomorphics. Senolytic agents, such as Dasatinib and Quercetin, selectively eliminate senescent cells, while senomorphic agents like Metformin and Rapamycin aim to modulate the senescence-associated secretory phenotype and restore cellular homeostasis. Despite these promising results, challenges remain, particularly in overcoming ocular drug delivery barriers. Nonetheless, the potential of targeting LECs senescence offers new therapeutic opportunities for cataract management. Collectively, these insights support a paradigm shift in cataract management. Rather than relying solely on surgical intervention, future strategies may emphasize biologically informed, disease-modifying, and preventive approaches that target cellular senescence.

## 1 Introduction

Cataract, defined as lens opacity, remains a leading cause of visual impairment worldwide. Despite the effectiveness of surgical intervention, cataracts remain the primary cause of blindness and moderate and severe vision impairment (MSVI), accounting for 45% and 39% of the global burden, respectively (Burton et al., 2021). The surgery approach has limitations, including postoperative complications (infection, posterior capsule opacification, intraocular lens dislocation, elevated intraocular pressure) and accessibility to medical interventions (Cicinelli et al., 2023; Terveen et al., 2022; Ting et al., 2024). These realities argue for developing disease-modifying therapies beyond surgery.

Understanding the molecular and cellular programs driving lens opacification is essential for developing therapies beyond surgery. Numerous studies have pointed to stress-induced senescence of lens epithelial cells (LECs) as a common mechanism across multiple cataract subtypes (Dong et al., 2024; Chen M., et al., 2022). In senescent LECs, antioxidant capacity is reduced, ion and water transport are disrupted, and orderly fiber-cell differentiation is impaired, all of which predispose the lens to opacity (Giannone et al., 2021; Huang et al., 2022). Cellular senescence in LECs disrupts key functions, contributing to lens opacity.

Throughout the lifespan, cumulative oxidative, metabolic, and mechanical stresses drive LECs toward a senescent state, characterized by functional decline and the secretion of pro-inflammatory factors (Liu H., et al., 2025). We propose that LECs senescence is a central pathogenic mechanism in cataractogenesis and represents a viable therapeutic target. In this review, we (i) discuss the primary stressors that induce LECs senescence, including oxidative damage, UV radiation, and metabolic disturbances; (ii) examine the molecular pathways, such as the p53/p21 axis and SASP, that contribute to the senescent phenotype in LECs; and (iii) highlight therapeutic strategies aimed at modulating LECs senescence, including senolytic agents to eliminate senescent cells and senomorphic agents to modulate their SASP secretions.

## 2 Cellular senescence

Cellular senescence is defined as a stable and irreversible arrest of the cell cycle, triggered by a variety of intrinsic and extrinsic stressors. These stressors commonly include hypoxia, nutrient deprivation, genotoxic insults, and elevated levels of oxidative stress (Kim et al., 2021; Dou et al., 2023; Rodier et al., 2009; Yousefzadeh et al., 2020; Cho and Kim, 2024). Under persistent sublethal stress, cells enter a non-proliferative yet metabolically active state that is distinct from reversible quiescence or terminal differentiation. The defining hallmarks of senescent cells include sustained cell-cycle arrest, the development of a senescence-associated secretory phenotype (SASP), accumulation of macromolecular damage, and disruptions in cellular metabolism (Victorelli et al., 2023; Wiley et al., 2016; Roh et al., 2023; Englund et al., 2023; López-Polo et al., 2024).

Among various forms of senescence, replicative senescence was the first to be described. In their seminal work, Hayflick and Moorhead demonstrated that human primary fibroblasts undergo irreversible growth arrest in the G1 phase following 40 to 60 population doublings—a phenomenon now widely known as the Hayflick limit (Hayflick and Moorhead, 1961). In addition to replicative senescence, other subtypes such as stress-induced premature senescence (SIPS) and programmed senescence have also been reported (Lucas et al., 2023; Kumari and Jat, 2021; Da Silva-Álvarez et al., 2019). These forms of senescence arise under different biological contexts and are driven by distinct upstream signals. SIPS, for instance, is typically triggered by oxidative stress or DNA damage without telomere shortening, while programmed senescence plays a crucial role during embryonic development and tissue remodeling (Muñoz-Espín et al., 2013; Chen et al., 2023; López-Polo et al., 2024).

Cellular senescence plays a dual role in tissue homeostasis, contributing to both beneficial processes, such as development and tissue remodeling, and detrimental effects when it becomes persistent or uncontrolled (de Magalhães, 2024; Feng et al., 2025). In adults, senescence acts as a tumor-suppressive barrier and coordinates tissue regeneration after injury (Galluzzi et al., 2016; Liu B., et al., 2025). During embryonic development, senescent cells regulate growth and patterning by secreting signaling molecules, ensuring normal tissue formation (Storer et al., 2013). Currently, however, there is limited research on the role of cellular senescence in lens development. In skin wound healing, senescent cells promote immune response and regeneration by secreting SASP factors, thereby facilitating the repair process (O'Reilly et al., 2024). However, when senescent cells accumulate due to immune dysfunction, they sustain a pro-inflammatory SASP, disrupting tissue homeostasis, impairing regeneration, and contributing to age-related diseases like cataracts (Sun et al., 2022; Coryell et al., 2021; Gaikwad et al., 2024; Qin et al., 2025). Therefore, elucidating the mechanisms of cellular senescence is essential for understanding age-associated diseases.

## 3 Physiological characteristics of the lens

### 3.1 Lens structure and physiological organization

The crystalline lens is an avascular structure located within the anterior segment of the eye, uniquely adapted to function in a low-oxygen microenvironment (Guo et al., 2023). Its primary physiological role is to precisely focus light onto the retina, a function that critically depends on its exceptional transparency and finely tuned refractive properties (Bassnett and Šikić, 2017). Structurally, the lens comprises three main components: an outer elastic capsule, a monolayer of LECs lining the anterior surface, and the underlying lens fiber cells (LFCs), which make up the majority of the lens. The lens capsule is a transparent basement membrane made of collagen IV, XVIII, and laminin, providing structural support and facilitating lens accommodation (Danysh and Duncan, 2009).

The anterior lens epithelium comprises a monolayer of metabolically active and proliferative cells that govern both lifelong lens growth and the maintenance of internal homeostasis (Liu et al., 2023). Functionally, these epithelial cells are spatially organized into three distinct zones. The central zone harbors relatively quiescent cells primarily involved in homeostatic maintenance, such as regulating ion transport and providing antioxidant defense (Sugiyama et al., 2008; Dahm et al., 2011). Encircling this is the germinative zone, characterized by active mitosis and serving as a reservoir for progenitor cells that support continuous lens growth. These newly generated cells migrate to the equatorial zone, where they withdraw from the cell cycle and undergo terminal differentiation into LFCs—a process essential for lens transparency and structural integrity (Dawes et al., 2018; Shaham et al., 2009).

This program of terminal differentiation, initiated at the equatorial region of the lens epithelium, proceeds through a highly coordinated sequence of events. As the epithelial cells elongate and differentiate into LFCs, they actively synthesize and accumulate large quantities of crystallin proteins while concurrently undergoing the systematic degradation of their nuclei, mitochondria, and other cytoplasmic organelles to minimize intra-lenticular light scattering (Tan et al., 2025; Cvekl and Eliscovich, 2021; Zhao Y. et al., 2018). These differentiated LFCs are then precisely arranged in tightly packed, concentric layers that envelop older fibers toward the lens core (Audette et al., 2017). Throughout this maturation process, LFCs progressively accumulate high levels of crystallins, which contribute to the formation of a dense paracrystalline cytoplasmic architecture. This ordered structure, together with the abundance of crystallins, provides the structural and biochemical foundation required for maintaining lens transparency and its precise refractive function (Quinlan and Clark, 2022).

The transparency of the lens relies on a lifelong process of tightly regulated cellular differentiation and migration. Central LECs, which are crucial for maintaining lens structure and function, have limited regenerative capacity. As a result, the long-term homeostasis of the lens is vulnerable to disruption by both intrinsic and extrinsic sources of molecular damage.

### 3.2 Functional roles of lens epithelial cells in homeostasis

The crystalline lens, as a complex, integrated system, maintains its transparency and long-term stability through a well-established antioxidant defense network. This system includes tight junctions between fiber cells and LECs, as well as the lens’s microcirculation, which allows efficient distribution of water, ions, metabolites, and antioxidants (Donaldson et al., 2023; Donaldson et al., 2017). These mechanisms are essential for protecting the lens from oxidative damage and maintaining its function (Giannone et al., 2021). Within this broader framework, LECs play a key role in maintaining lens homeostasis. They possess strong antioxidant capabilities and also regulate the intra-lenticular physicochemical environment and material exchange (Sorte Gawali et al., 2024; Ma et al., 2023). Additionally, LECs serve as the primary site for metabolic and biosynthetic activity in the lens, further contributing to its overall function and stability (Liu et al., 2023).

First, LECs regulate the intra-lenticular physicochemical environment and material exchange. Through membrane-bound ion pumps (notably Na^+^/K^+^-ATPase) and aquaporins, LECs actively sustain the characteristic ionic gradients (low Na^+^, high K^+^, low Ca^2+^) and water content of the lens, which are fundamental to its refractive index and transparency (Donaldson et al., 2024; Donaldson et al., 2023). In addition, LECs establish extensive intercellular communication with LFCs via gap junctions, forming the basis of the lens’s microcirculation system. This system enables efficient intra-lenticular transport of water, ions, metabolites (e.g., glucose), and antioxidants (e.g., glutathione) (Beyer et al., 2022; Liu et al., 2023). Second, LECs serve as the primary site for metabolic and biosynthetic activity in the lens. Most biomolecules essential for lens maintenance and fiber cell differentiation are synthesized or processed within LECs. This includes: (1) energy metabolism, primarily via glycolysis to generate ATP (Zahraei et al., 2022; Giannone et al., 2021); (2) lipid metabolism, which produces cholesterol and other membrane lipids (Timsina et al., 2024; Cenedella, 1982; Okada et al., 1971); and (3) protein metabolism, involving the synthesis of key enzymes and structural proteins (McAvoy, 1980; Andley et al., 1998). LECs also play a key role in the lens’s primary antioxidant defense by producing high levels of glutathione (GSH) and expressing various enzymatic systems, including catalase, superoxide dismutase (SOD), and thioredoxin/glutaredoxin pathways (Zhou et al., 2021; Rajkumar et al., 2008).

Together, these functions constitute a multilayered antioxidant defense system. LECs not only generate antioxidant molecules but also facilitate their distribution to deeper lens regions through the lens microcirculation, while maintaining an ionic environment essential for crystallin stability. Through coordinated regulation of transport, metabolism, and biosynthesis, LECs protect the lens from cumulative oxidative damage and preserve long-term transparency. With aging, however, these protective mechanisms gradually decline, leading to increased oxidative stress, cellular senescence, and ultimately lens opacity. Additionally, the distinctive biological characteristics of the lens position it not only as a specialized ocular organ but also as a model for studying cellular senescence and aging. Its avascular nature minimizes confounding influences from systemic circulation, enabling clearer dissection of intrinsic aging mechanisms. Its simple and well-ordered structure provides a traceable, linear sequence of cellular changes. Furthermore, lens transparency, a direct and quantifiable functional outcome, offers a unique phenotypic readout for evaluating age-related decline and the efficacy of anti-aging interventions.

## 4 Molecular pathogenesis of LECs senescence

### 4.1 Cellular responses and the establishment of senescence in LECs

Although most anterior LECs are quiescent or slowly cycling under physiological conditions, stress disrupts lens epithelial homeostasis and converts this restrained state into a senescent program by activating DNA damage and redox checkpoints. Key features of LEC senescence include cell-cycle arrest, development of SASP, and autophagy dysfunction, which are closely linked to the onset and persistence of senescence.

#### 4.1.1 Cell cycle arrest and proliferation inhibition

Stable and virtually irreversible cell-cycle arrest represents the most fundamental hallmark of cellular senescence. It is widely recognized that senescent cells primarily arrest in the G1 phase of the cell cycle, thereby preventing the replication of damaged DNA. However, some senescent cells may also undergo arrest in the G2 phase to block mitotic entry in the presence of persistent genomic damage (Kumari and Jat, 2021; Ogrodnik et al., 2024). Cyclin-dependent kinases (CDKs) are serine/threonine kinases that form complexes with specific cyclins. These CDK–cyclin complexes promote cell cycle progression by phosphorylating critical substrates required for DNA replication and entry into mitosis (Pellarin et al., 2025). Proper activation and regulation of CDK activity is essential for orderly cell cycle transitions, particularly the progression from G1 to S phase and from G2 to M phase (Pellarin et al., 2025; Sunada et al., 2021). In response to various cellular stressors, LECs activate canonical tumor-suppressive pathways, notably the p53/p21 and p16^INK4a^/Rb axes, which enforce a stable blockade of cell cycle progression (Liu H., et al., 2025). A range of signaling pathways converge to modulate this arrest program.

Cell-cycle arrest and proliferative inhibition represent the most prominent hallmarks of LECs senescence. In our previous studies, we demonstrated that heme oxygenase-1 (HO-1) delays LECs senescence by enhancing the antioxidant defense system and suppressing activation of the p53/p21 signaling pathway (Wang et al., 2023). Additionally, biliverdin reductase A (BVRA) exerts cytoprotective effects through multiple mechanisms. It catalyzes the conversion of biliverdin (BV) to bilirubin (BR), upregulates the expression of HO-1, and activates the ERK/Nrf2 signaling pathway. Together, these actions enhance the antioxidant capacity of LECs, thereby delaying the onset of cellular senescence. Moreover, BVRA has been shown to inhibit the activation of the p21 and p16, further contributing to the suppression of stress-induced senescence (Huang et al., 2022). In addition, sirtuin 1 (SIRT1), a key NAD^+^-dependent deacetylase, has been shown to exert anti-senescent effects in LECs. Under oxidative stress, the downregulation of SIRT1 enhances the acetylation of p66Shc, thereby promoting its activation and mitochondrial translocation. This process exacerbates mitochondrial ROS production, establishing a vicious cycle of oxidative damage (Liu H., et al., 2025). Mitochondrial dysfunction not only directly impairs cellular energy metabolism but also activates cell cycle inhibitory pathways, ultimately driving cells into a senescent state (Liu H., et al., 2025). The SIRT1/Nrf2 signaling axis has also been shown to delay cellular senescence by enhancing the intrinsic antioxidant defenses of LECs (Chen Q., et al., 2022). Moreover, SIRT1 has been shown to exert anti-senescent effects by inhibiting the FoxO1/TLR4 signaling pathway (Jiang H., et al., 2025). In diabetic cataract patients, aberrant N6-methyladenosine (m^6^A) modification has been shown to promote the degradation of SIRT1 mRNA, leading to decreased SIRT1 expression. This downregulation subsequently results in the upregulation of the p53/p21 signaling pathway, thereby accelerating cellular senescence in LECs (Dong et al., 2024). In addition, several emerging molecular pathways have recently been identified. Loss of FYVE and FYCO1 has been shown to attenuate oxidative stress-induced senescence by suppressing the PAK1/p21 signaling pathway (Chen S., et al., 2024). In cataract models induced by elevated uric acid, uric acid promotes chronic inflammation by activating the NLRP3 inflammasome. This inflammatory cascade subsequently triggers the p53/p21 pathway, thereby inducing senescence in LECs (Lin et al., 2024). Mechanical strain may also contribute to the senescence of LECs. Under tensile stress, activation of the mechanosensitive ion channel PIEZO1 leads to calcium influx, which in turn triggers the expression of senescence-associated genes (Chen L., et al., 2024). Notably, p21 expression is not always regulated through the canonical p53-dependent pathway. Evidence suggests that p53-independent mechanisms of p21 regulation also exist. For instance, Heat Shock Factor 4 (HSF4) can suppress p21 transcription by recruiting the histone methyltransferase EZH2 to the p21 promoter region, thereby enhancing H3K27me3 modification and silencing p21 expression (Cui et al., 2021).

#### 4.1.2 SASP activation and microenvironmental modulation in senescent LECs

Beyond stable cell-cycle arrest, one of the most prominent features of senescent cells is the development of a complex and pro-inflammatory secretory program known as the senescence-associated secretory phenotype (SASP). The SASP encompasses a wide array of bioactive molecules, including cytokines, chemokines, matrix metalloproteinases (MMPs), and growth factors (Gorgoulis et al., 2019; Cao et al., 2025). These molecules exert their effects either through autocrine reinforcement of senescence or by modulating the microenvironment via paracrine signaling pathways. Functionally, the SASP constitutes the principal mechanism through which senescent cells exert their effects on the surrounding tissue, actively remodeling the microenvironment and contributing to both physiological and pathological processes (Wang et al., 2024). Although comprehensive studies of SASP in cataract remain limited, existing evidence has observed elevated expression of canonical SASP factors in LECs senescence models, which suggests that SASP may play a meaningful role in LECs aging. Once senescence is established, the SASP can actively reshape the tissue microenvironment, impair the function of neighboring non-senescent LECs, and thereby accelerate the propagation of senescence within the lens epithelium.

Emerging data also implicate SIRT1 in the regulation of SASP production. Specifically, treatment of senescent LECs with resveratrol, a pharmacological activator of SIRT1, has been shown to reduce the secretion of multiple SASP factors, including IL-1β, IL-6, CXCL8, MMP1, and TGF-β. Resveratrol enhances the antioxidant capacity of LECs by activating the SIRT1/Nrf2 signaling pathway, thereby preventing the onset of cellular senescence (Chen Q., et al., 2022). HSF4 has also been implicated in the regulation of SASP (Cui et al., 2021). In addition, another study reported an upregulation of IL-1 expression in senescent LECs. Although this study did not explicitly define the response as SASP, the increased expression of IL-1β, a core component of the SASP, further supports the notion that inflammatory secretory activity is a prominent feature of LEC senescence (Lin et al., 2024). Similarly, TGF-β1 and MMP-9 may also function as components of the SASP, contributing to the senescence process of LECs (Yan et al., 2019).

#### 4.1.3 Autophagy dysfunction

Autophagy is an evolutionarily conserved lysosome-mediated catabolic process that plays a central role in maintaining cellular homeostasis by degrading and recycling damaged organelles, misfolded proteins, and other cytoplasmic components (Gómez-Virgilio et al., 2022). And under stress, autophagy acts as a crucial adaptive mechanism that allows cells to survive nutrient deprivation, oxidative insults, and other damaging stimuli (Kaushik et al., 2021; Ryteret al., 2013; He, 2022). In the context of lens biology, increasing evidence suggests that autophagy may influence the susceptibility of LECs to senescence, particularly under conditions of chronic stress such as oxidative damage or metabolic imbalance. Our previous research demonstrated that HO-1 delays oxidative stress–induced senescence by promoting the nuclear translocation of transcription factor EB (TFEB), thereby enhancing autophagic flux and lysosomal function (Wang et al., 2023). Similarly, SIRT1 has been shown to attenuate cellular senescence through the regulation of autophagic activity. The SIRT1 activator SIRT1720 increases the number of autophagosomes and restores autophagic flux by upregulating Beclin1 and LC3II/I and downregulating p62. This leads to the reversal of P53 and P21 upregulation (Dong et al., 2024). FYCO1, an autophagy adaptor protein, also contributes to the maintenance of autophagic flux and exerts anti-senescent effects (Chen S., et al., 2024). Conversely, impaired autophagic flux has been linked to the activation of the p38 MAPK/p53 signaling axis, thereby promoting the onset of senescence (Xu et al., 2018). These findings collectively underscore the pivotal role of functional autophagy in maintaining LECs homeostasis under stress conditions.

### 4.2 Molecular triggers of LECs senescence

**FIGURE 1:**
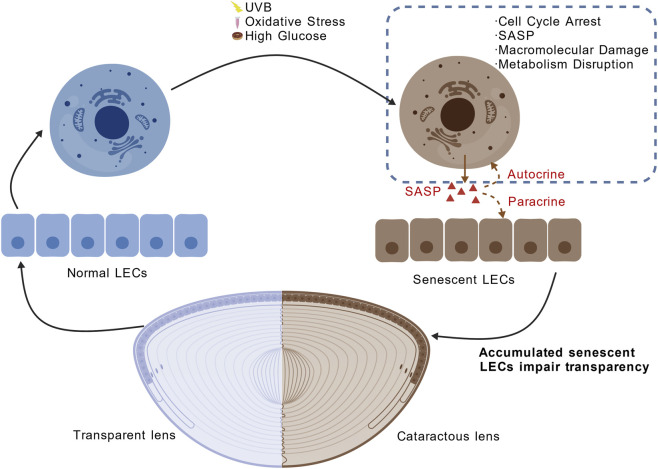
Senescence-Inducing Stress in LECs and Its Role in Cataractogenesis. Chronic UVB and metabolic stress activate DNA damage response and increase mitochondrial ROS in lens epithelial cells. These changes lead to cellular senescence and SASP. Loss of epithelial homeostatic functions and paracrine dysfunction disrupt lens transparency and result in cataract. The pattern was created with BioGDP.com (Jiang S., et al., 2025).

The pathogenesis of cataract fundamentally stems from the progressive dysfunction of LECs, in which cellular senescence plays a central role. A wide range of chronic or cumulative stressors, such as oxidative stress, ultraviolet B (UVB) radiation, and high-glucose environments, can induce sublethal cellular damage that drives LECs into a senescent state (Zhang, 2024; Dong et al., 2024). These cells exhibit hallmark features such as stable cell-cycle arrest and acquisition of SASP, which impair their regulatory functions and promote a pro-inflammatory microenvironment (Wang et al., 2023; Wang Y., et al., 2022) (Figure 1).

#### 4.2.1 Oxidative stress and phototoxicity

Among various environmental stressors, UVB radiation is recognized as one of the most prominent exogenous sources of oxidative stress contributing to lens aging. UVB, with a wavelength range of 280–315 nm, can penetrate the cornea and reach the anterior capsule of the lens, exerting direct and cumulative phototoxic effects on LECs (Hamba et al., 2021; MacFarlaneet al., 2024). A growing body of epidemiological and experimental evidence has also linked UVB to an increased risk of cortical age-related cataracts (McCarty et al., 2000; Li et al., 2021). UVB exerts its deleterious effects on LECs primarily through the induction of oxidative stress and DNA damage. At the cellular level, UVB exposure can cause DNA double-strand breaks (DSBs), thereby activating the DNA damage response (DDR) pathway (Mesa and Bassnett, 2013; Liu et al., 2021). Concurrently, UVB-induced photoexcitation leads to the overproduction of ROS, which disrupts redox homeostasis and impairs mitochondrial function. With advancing age, the lens exhibits a diminished capacity to counteract oxidative stress, as evidenced by declining GSH levels and reduced activity of antioxidant enzymes. This impairment renders senescent LECs particularly susceptible to prolonged oxidative damage induced by UVB radiation (Bejarano et al., 2023; Pescosolido et al., 2016). Taken together, these cumulative insults can drive cells into a state of irreversible cell-cycle arrest accompanied by persistent expression of senescence-associated phenotypes, including elevated pro-inflammatory cytokine production. In addition to exogenous ultraviolet radiation, LECs are chronically exposed to endogenous oxidative stress arising from intrinsic metabolic activity throughout lifespan. GSH is significantly reduced in aged individuals and in cataractous lenses (Sweeney and Truscott, 1998; Kamei, 1993). Similarly, enzymatic antioxidant systems such as SOD and catalase show diminished activity with age, compromising the clearance of ROS (Fecondo and Augusteyn, 1983; Rajkumar et al., 2008). Our previous work also identified a marked downregulation of BVRA in cataractous lens tissue. BVRA enzymatic activity was found to decline progressively with cellular senescence (Huang et al., 2022). This dual loss of expression and function may further impair the capacity of LECs to defend against oxidative insults.

#### 4.2.2 Metabolic stress and glucotoxicity

Metabolic dysregulation, particularly glucotoxicity induced by chronic hyperglycemia, represents a potent and prototypical source of cellular stress in the pathophysiological aging of the lens. In diabetic cataracts, LECs are chronically exposed to a pathological microenvironment characterized by increased oxidative stress and osmotic imbalance (Guo et al., 2023). This adverse cellular context serves as a key upstream factor driving LECs dysfunction and promoting premature senescence. Mechanistically, high glucose activates multiple stress-associated intracellular pathways. One of the most well-characterized is the polyol pathway, which becomes hyperactivated under hyperglycemic conditions, leading to sorbitol accumulation and consequent osmotic stress (Thorne et al., 2024). Simultaneously, non-enzymatic glycation of proteins and lipids accelerates the formation of advanced glycation end products (AGEs), which bind to their receptor, the receptor for advanced glycation end products (RAGE), and perpetuate inflammatory and oxidative signaling cascades (Cooksley et al., 2024).

#### 4.2.3 Other stress factors

Beyond metabolic and oxidative stress, LECs are also subject to a range of other physiological and pathological stressors. Several studies have demonstrated that mechanical stretch activates stress-responsive signaling cascades, inducing ROS generation, cytoskeletal remodeling, and the release of inflammatory mediators (Liang et al., 2019; Chen L., et al., 2024). These findings highlight the potential for biomechanical forces to serve as upstream triggers of cellular senescence in lens epithelium. In addition, systemic metabolic abnormalities, such as hyperuricemia and dysregulated lipid metabolism, can lead to the accumulation of metabolic waste products in the aqueous humor, thereby altering the physicochemical properties of the lens microenvironment (Lin et al., 2024; Wang J., et al., 2022). Recent studies have suggested that certain metabolic byproducts may directly or indirectly trigger oxidative stress in LECs. For example, elevated concentrations of uric acid have been shown to activate the NLRP3 inflammasome, contributing to LECs dysfunction and the secretion of SASP (Lin et al., 2024). The pathological connection between systemic metabolic waste and LECs functional integrity may represent a novel mechanistic axis for understanding how systemic metabolic risk factors contribute to cataractogenesis.

#### 4.2.4 Animal models for studying LEC senescence in cataract formation

Various animal models have been developed to study the role of LEC senescence in cataract formation. One of the most widely used models involves UVB radiation, which induces oxidative stress and DNA damage in LECs. UVB exposure causes the accumulation of ROS and triggers senescence pathways in LECs, leading to the development of cataracts (Liu H., et al., 2025; Chen S., et al., 2024). Besides, D-galactose and sodium selenite are also used to construct cataract models by inducing LEC senescence through oxidative stress (Wang Y., et al., 2022; Chen Q., et al., 2022). In addition to induced models, naturally aged animals are also used to study cataractogenesis. These models provide a more physiologically relevant system in which LEC senescence naturally accumulates over time, leading to gradual lens opacity (Chen M., et al., 2022). In addition, increased LEC senescence and early cataract onset have been observed in genetic knockout mice, such as those with YAP conditional knockout or AQP5 deficiency, both of which promote LEC senescence (Zhang K., et al., 2023; He et al., 2019). Together, these animal models have been pivotal in advancing our understanding of the molecular mechanisms that drive LEC senescence in cataract formation.

### 4.3 Heterogeneity of senescent LECs

Cellular senescence is increasingly recognized as a heterogeneous phenomenon, encompassing diverse subtypes that differ in their secretory profiles, metabolic states, and stress-response signatures. Recent advances in single-cell transcriptomics and proteomics have revealed such heterogeneity in fibroblasts, epithelial cells, and immune cells. For instance, dermal fibroblasts exhibit heterogeneous senescent subtypes that can be distinguished according to their functional attributes (Kamat et al., 2025; Hughes et al., 2025; Zou et al., 2021). In contrast, studies specifically addressing the heterogeneity of senescent LECs remain limited. A recent study utilizing scRNA-seq functionally categorizes human LECs into four subtypes (eLECs, mLECs, lLECs, and TACs), and demonstrates that although the proportional distribution of these subtypes remains stable between aged and younger individuals, their transcriptional profiles are significantly altered with age. These age-related changes include downregulation of cell adhesion functions and upregulation of oxidative stress–related pathways, suggesting that LECs subtype-specific vulnerability may contribute to cataractogenesis (Gu et al., 2025). This work lays a critical foundation for future efforts to construct a senescence atlas of the lens at single-cell resolution. Identifying and functionally characterizing senescent LECs subtypes could refine our understanding of lens aging and uncover novel therapeutic entry points for delaying or reversing cataract development.

## 5 Anti-senescence interventions in cataract: therapeutic strategies and translational challenges

A deeper understanding of the molecular mechanisms underlying cellular senescence in cataract has unveiled promising new therapeutic opportunities aimed at pharmacologically delaying or potentially halting the progression of this age-related disease. These therapeutic interventions can be broadly categorized into two principal classes, senolytics and senomorphics. Senolytics are designed to selectively eliminate senescent cells by exploiting their unique survival dependencies (Suda et al., 2024). Senomorphics aim to suppress the SASP without necessarily inducing cell death, thus preserving tissue microenvironment (Barnes, 2025). Both approaches represent distinct but complementary avenues for mitigating the pathological consequences of senescent cells accumulation in the aging lens. Importantly, these emerging therapies offer a conceptual shift from solely surgical intervention toward a more mechanistic, disease-modifying approach to cataract treatment.

### 5.1 Anti-senescence therapeutic strategies

#### 5.1.1 Senolytics

Senolytics refer to a class of small-molecule compounds that selectively induce apoptosis in senescent cells while sparing healthy, proliferating counterparts (Lagoumtzi and Chondrogianni, 2021). Key components of these survival pathways include anti-apoptotic members of the BCL-2 protein family and signaling regulators such as the p53/p21 axis, which together help maintain the viability of senescent cells by inhibiting programmed cell death (Rad and Grillari, 2024; Alum et al., 2025). By selectively disrupting critical nodes within these pathways, senolytic agents effectively dismantle the molecular support systems that sustain senescent cells, thereby reactivating intrinsic apoptotic mechanisms and promoting their clearance.

The concept of senolytic therapy was first proposed by Kirkland and colleagues, who identified the vulnerability senescent cell anti-apoptotic pathways as a therapeutic target. Their work demonstrates that the combination of Dasatinib (a tyrosine kinase inhibitor) and Quercetin (a natural flavonoid) could synergistically eliminate senescent cells without harming normal cells (Zhu et al., 2015). Since then, several other senolytics have been developed. Notably, Navitoclax (ABT-263), a BCL-2 family inhibitor originally designed as an anti-cancer drug, exhibits potent senolytic activity by specifically disrupting Bcl-2/Bcl-xL interactions with pro-death proteins, thereby neutralizing their anti-apoptotic function and triggering senescent cell death (Mylonas and Ferenbach, 2024). In senescent cells, FOXO4 exerts an anti-apoptotic effect by binding to and sequestering the nuclear protein p53, thereby preventing the initiation of apoptosis (Bourgeois and Madl, 2018). Disruption of this interaction has been shown to induce selective apoptosis of senescent cells. For instance, the synthetic peptide FOXO4-D-Retro-Inverso (FOXO4-DRI) competitively inhibits the FOXO4–p53 interaction, thereby reactivating p53-mediated apoptotic signaling and functioning as a senolytic agent (Baar et al., 2017). Additionally, Fisetin, a naturally occurring flavonoid, has also been reported to reduce senescent cell burden in multiple preclinical models (Syed et al., 2016; Zhang L., et al., 2023).

#### 5.1.2 Senomorphics

Senomorphics aim to modulate the secretion of the SASP in senescent cells by targeting key signaling pathways responsible for its induction. The goal is to convert senescent cells from a pro-inflammatory, tissue-damaging state into a more quiescent or functionally inert condition. This approach may offer a particularly favorable safety profile in non-regenerative tissues such as the lens epithelium, where cellular clearance could lead to loss of barrier function or impaired structural integrity due to the limited proliferative capacity of neighboring cells.

Rapamycin (also known as sirolimus) is a macrolide compound originally isolated from *Streptomyces hygroscopicus* (Vézina et al., 1975). It functions by inhibiting TORC1, thereby reducing the phosphorylation of its downstream effectors S6K and 4E-BP, which are critical for translational control of SASP components (Bjedov and Rallis, 2020; Choo et al., 2008). Numerous studies have demonstrated that Rapamycin can suppress both cellular senescence and the expression of SASP, thereby alleviating the chronic inflammatory burden associated with aging (Hoff et al., 2022; Wang et al., 2017). Additionally, Metformin has recently garnered attention for its broader pharmacological properties beyond glycemic control (Abou Zaki and El-Osta, 2024). Among these is its emerging role in ameliorating age-related functional decline. It has been shown to suppress the NF-κB signaling cascade by inhibiting the phosphorylation of IκB and its upstream kinases IKKα/β, thereby attenuating the production of pro-inflammatory SASP factors (Moiseeva et al., 2013). In addition, low-dose Metformin activates the Nrf2–GPx7 signaling axis by promoting Nrf2 nuclear accumulation and upregulating GPx7 expression to delay cellular senescence (Fang et al., 2018). NF-κB is both a central transcriptional regulator of SASP and a pivotal driver of the senescence program itself (Schlett et al., 2023). SR12343, for example, exerts its anti-inflammatory and anti-senescent effects by disrupting the interaction between IKKβ and NEMO, thereby blocking NF-κB activation induced by tumor necrosis factor-α (TNF-α) and lipopolysaccharide (LPS) stimulation (Zhao J. et al., 2018; Zhang et al., 2021). The mitogen-activated protein kinase (MAPK) signaling pathway is a classical and evolutionarily conserved cascade involved in transducing various extracellular stimuli into cellular responses. Upon activation, p38 MAPK translocates these stress signals to the nucleus, where it promotes cell cycle arrest and the synthesis of SASP factors (Odawara et al., 2024). Several p38 MAPK inhibitors, including SB203580, UR13756, and BIRB796, have been shown to reduce SASP secretion and mitigate its deleterious paracrine pro-senescent effects (Kuk et al., 2024; Alimbetov et al., 2016). Moreover, inhibition of MK2 kinase, a key downstream effector of p38 MAPK, has similarly been demonstrated to suppress SASP production (Alimbetov et al., 2016).

### 5.2 Anti-senescence interventions in cataract

As previously discussed, the accumulation of senescent LECs, together with their detrimental effects arising from functional decline and the secretion of SASP factors, plays a central role in the pathogenesis of cataract. Recent studies have shown that selectively eliminating senescent LECs or pharmacologically suppressing their SASP output can help preserve lens transparency and delay cataract progression (Table 1). These findings underscore the pathogenic importance of LECs senescence and provide a strong rationale for targeting senescent cells as a therapeutic strategy.

**TABLE 1:** Mechanisms and efficacy of anti-senescence drugs in cataract treatment.

Agent	Mechanism of action	Effect	*In vivo* efficacy	References
Dasatinib and Quercetin	Induce cell death in senescent cells	Reduce the LEC senescence burden	Alleviate cataract progression	Wang Y., et al. (2022)
Rapamycin	Inhibit mTOR	Inhibit the expression of SASP-related cytokines	Slow cataract progression	Wang Y., et al. (2022)
Metformin	Upregulat SIRT1 expression, activate AMPK signaling pathway, inhibite LARP1	Restore autophagic function, improve mitochondrial function, delay senescence in LECs	Alleviate cataract development	Fu et al. (2024), Chen et al. (2021), Xu et al. (2018), Qi et al. (2025)
Resveratrol	Aactivate SIRT1/Nrf2 signaling pathway	Mitigate cellular senescence and delay the onset of cataract	Reduce the lens opacity	Chen Q. et al. (2022)

As a well-established anti-senescence strategy, the combination of Dasatinib and Quercetin (D + Q) has been demonstrated to clear senescent cells through the induction of apoptosis (Liu J., et al., 2025). Additionally, this D + Q regimen has been shown to mitigate the progression of D-galactose-induced cataracts by reducing cellular senescence in LECs. Following D + Q treatment, aged rats exhibited decreased expression of senescence markers such as p16 and γH2AX, along with significantly improved indicators of lens transparency compared to untreated controls. These findings highlight the therapeutic potential of D + Q in mitigating cataract progression (Wang Y., et al., 2022). Similarly, Metformin has gained increasing support from both *in vivo* and *in vitro* studies for its potential application in cataract therapy. In diabetic cataract models, Metformin delays cataract development by upregulating SIRT1 expression, restoring autophagic function in LECs, and alleviating cellular senescence (Dong et al., 2024; Fu et al., 2024). Furthermore, Metformin also prevents premature senescence in LECs by improving mitochondrial function (Xu et al., 2018). Beyond diabetic cataracts, Metformin has been shown to exerts anti-senescence effects in models of chronic oxidative stress and in naturally aged mice, primarily through the activation of the AMPK signaling pathway (Zhang et al., 2020; Chen M., et al., 2022; Chen et al., 2021). Recent evidence suggests that La-related protein 1 (LARP1) may mediate part of Metformin’s anti-senescence effects. By inhibiting LARP1, Metformin reduces the formation of stress granules and ameliorates mitochondrial dysfunction, thereby contributing to cellular homeostasis (Qi et al., 2025). Moreover, Rapamycin has been shown to reduce cataract progression, with improved lens clarity and reduced cataract severity in treated rats on day 21 compared to the vehicle group. However, these effects were not sustained by day 28 (Wang Y., et al., 2022). In addition, Resveratrol, an activator of SIRT1, has been shown to enhance the antioxidant capacity of LECs by activating the SIRT1/Nrf2 signaling pathway. This activation mitigates cellular senescence and delays the onset of cataract formation (Chen Q., et al., 2022).

Overall, growing evidence suggests that targeting cellular senescence can effectively slow cataract progression by preserving lens epithelial cell function and maintaining lens transparency. These findings highlight the potential of anti-senescence therapies in cataract intervention and provide new perspectives for clinical management of the disease.

### 5.3 Translational challenges in senescence-targeted cataract therapies

Although senescence-targeted strategies offer promising avenues for pharmacological intervention in cataract, their successful translation from bench to bedside remains hindered by several significant scientific and technical obstacles. One of the most important factors is the issue of the administration route. The corneal epithelium, conjunctival vasculature, and tear-film dynamics collectively contribute to poor drug penetration and rapid clearance, often resulting in ocular bioavailability below 5% (Onugwu et al., 2023; Yang and Lockwood, 2022). Despite the fact that invasive delivery routes, including intravitreal or intracameral injections, can bypass these anterior segment barriers, their use is associated with risks such as infection, intraocular pressure fluctuations, and other complications (Maruyama-Inoue et al., 2021). Thus, the ideal route of administration is topical eye drops, which provide a non-invasive approach but are severely limited by pharmacokinetic constraints. Recent advancements in nanotechnology have enabled the development of targeted drug delivery systems, such as nanoparticle-based eye drops, that can effectively penetrate ocular barriers and directly deliver therapeutics to the lens. Examples of these systems include ceria nanoparticles which target mitochondrial oxidative stress, EGCG-Zn nanoparticles targeting intracellular oxidative stress, and aromatized lipid nanoparticles (LNPs) which deliver mRNA to the lens (Jiang L., et al., 2025; Wang et al., 2025; Song et al., 2025). These advancements highlight the need for developing novel delivery systems, such as nanocarriers or permeability enhancers, to overcome ocular barriers and ensure sustained therapeutic effects within the lens.

## 6 Conclusion and future perspectives

The lens possesses a robust anti-senescence system, supported by tight junctions, microcirculation, and antioxidants. Furthermore, growing evidence emphasizes the pivotal role of senescent LECs in cataractogenesis. Senescent LECs contribute to lens opacification through both the loss of their physiological functions and the detrimental effects of SASP, which disrupts local homeostasis. Cataract should no longer be seen solely as a condition needing surgery. While surgery is effective and curative, attention must also be given to issues like post-operative complications and the lack of timely medical access. Moreover, cataracts involve underlying molecular changes that could potentially be targeted with pharmacological treatments, offering a broader approach to management. It also involves molecular changes that could be targeted with pharmacological treatments. Emerging strategies such as senolytics and senomorphics offer promising avenues for delaying or preventing cataract progression by directly targeting senescent cells and their downstream effects.

Although LECs are predominantly quiescent, stress factors can drive their transition to a senescent state. This process is characterized by cell cycle arrest, DNA damage, and impaired autophagic flux. Senescent LECs exhibit SASP, inducing chronic inflammation and a pro-fibrotic microenvironment. Additionally, activation of stress-responsive pathways reinforces the senescent phenotype. These alterations disrupt lens homeostasis, contributing to cataract formation and progression. In addition, although most LFCs are in the postmitotic stage, they may still exhibit senescence characteristics under stress conditions. Studies have shown protein expression differences between LFCs in different regions of the lens, suggesting that LFCs may undergo age-related changes (Cantrell and Schey, 2021). However, research on LFCs and senescence is currently limited and requires further investigation. Additionally, recent studies have shown the presence of immune cells in the anterior segment of the lens (Gu et al., 2025). Based on the role of immune cells in other tissues in clearing senescent cells, it is speculated that these cells may be involved in the clearance of senescent LECs. However, their exact function remains to be further explored. Recent research also found an increased proportion of senescent LECs in cataract patients, suggesting that the accumulation of senescent LECs may be a contributing factor to cataract formation (Ye et al., 2022). Besides, although various animal models have been utilized to study the role of LEC senescence in cataract formation, these models can only partially replicate clinical conditions, and more refined models are needed for further exploration.

Realizing the therapeutic potential of senescence-targeted interventions for cataract requires overcoming several critical scientific challenges. Foremost among these is the need to characterize the heterogeneity of senescent LECs using single-cell technologies. Such analyses are essential for identifying pathogenic subpopulations that contribute to lens dysfunction. This knowledge would enable the development of precision strategies capable of selectively eliminating deleterious senescent cells while preserving those that retain functional activity. Based on these insights, further approaches—such as targeted clearance or phenotypic reprogramming—can be designed to better accommodate the lens’s unique non-regenerative architecture and its long-term homeostatic requirements. While senescence-targeted strategies such as senolytics and senomorphics offer promising therapeutic avenues for cataract treatment, a significant challenge remains in developing non-invasive methods for delivering these therapies to the eye. The ocular barrier presents substantial obstacles to efficient drug delivery. Although topical eye drops provide a non-invasive option, their effectiveness is limited by pharmacokinetic constraints. The development of advanced delivery systems, such as nanocarriers and permeability enhancers, is crucial for overcoming these barriers and maintaining therapeutic drug concentrations in the aqueous humor. Therefore, future research must prioritize enhancing the ocular bioavailability of senescence-targeted therapies to effectively prevent or delay cataract progression without the need for invasive procedures.

In summary, cataracts remain a major global cause of blindness, and while surgical intervention has long been the standard treatment, recent advances in understanding the role of LECs senescence offer new opportunities for disease-modifying therapies. Targeting the cellular senescence process through senolytic and senomorphic strategies holds great promise for delaying or even preventing cataract progression. These therapeutic approaches aim to eliminate senescent cells or modulate their detrimental secretory phenotype, thereby preserving lens transparency and function. Moving forward, a combined approach integrating pharmacological treatments and advanced delivery technologies could revolutionize cataract management, shifting the focus from surgical solutions to preventive, biologically informed therapies.
